# TWO PATHS TO COMMUNITY CARE FOR OLDER ADULTS: KOREA AND SINGAPORE’S LONG-TERM CARE EVOLUTION

**DOI:** 10.1093/geroni/igae098.1670

**Published:** 2024-12-31

**Authors:** Jeremy Lim-Soh, Angelique Chan, Aejung Yoo, Dahye Kim, Yugyeong Eo, Jae Woo Choi, Wan Chen Graham, Loong Mun Wong

**Affiliations:** Duke-NUS Medical School, Singapore, Singapore; Duke-NUS Medical School, Singapore, Singapore; Health Insurance Research Institute, National Health Insurance Service, Wonju, Kangwon-do, Republic of Korea; National University of Singapore, Singapore, Singapore; Center for Social Services Research, Korea Institute for Health and Social Affairs, Sejong, Chungchongnam-do, Republic of Korea; Health Insurance Research Institute, National Health Insurance Service, Wonju, Kangwon-do, Republic of Korea; Agency for Integrated Care, Singapore, Singapore; Agency for Integrated Care, Singapore, Singapore

## Abstract

Aging populations pose significant challenges to institutional Long-Term Care systems. Community Care services, which provide professional support for functional limitations, chronic diseases, or cognitive impairment to community-dwelling older adults, have emerged as a potentially cost-effective solution that can support aging-in-place. However, differing implementations of Community Care across societies can affect older adults’ health and quality of life. We conduct a comparative case study of the evolution of Long-Term Care policy in Korea and Singapore towards Community Care. Both societies exhibit similar demographic trends—rapid aging, ultra-low fertility, and declining old-age support ratios—coupled with Confucian cultural ideals. However, their welfare systems differ historically; Korea took a welfare state approach emphasizing institutional care, and Singapore leaned towards neoliberalism with a reliance on informal care. While both societies are developing various Community Care programs including home-based nursing, day care centers, and assisted living, we find significant differences in the way that Community Care is planned, funded, and implemented. Moreover, the challenges faced depend on cultural context and welfare system. Based on these insights, we discuss the compatibility of Community Care with different sociocultural settings and policy ecosystems, concluding with potential implications for other societies struggling with population aging and LTC sustainability.

